# Pulmonary Manifestations of Inflammatory Bowel Disease

**DOI:** 10.7759/cureus.14216

**Published:** 2021-03-31

**Authors:** Nicole Tejeda Taveras, Arlenys Rivera Martinez, Ram Kumar, Amna Jamil, Besham Kumar

**Affiliations:** 1 Internal Medicine, Universidad Iberoamericana, Santo Domingo, DOM; 2 Internal Medicine, Universidad Iberoamericana, Santo Domingo, DOM; 3 Internal Medicine, Chandka Medical College, Larkana, PAK; 4 Internal Medicine, Jinnah Postgraduate Medical Centre, Karachi, PAK

**Keywords:** ibd, inflammatory bowel disease, pulmonary manifestations

## Abstract

The frequency of extraintestinal manifestations of inflammatory bowel disease (IBD) is observed in most of the patients. Extraintestinal manifestations of IBD have been implicated in all the anatomic sites of the pulmonary tree, and include airway inflammation of the bronchi and bronchioles and parenchymal inflammation. There is a wide range of pulmonary manifestations in IBD, and most of them look a lot like other diseases. Doctors should keep a track of these conditions to avoid unnecessary complications and to provide an early diagnosis with correct and efficient treatment. IBD-related respiratory disorders are treated depending on the patient, but in most of them, steroids are generally chosen first. Steroids, both inhalational and systemic, are the primary approach. Antibiotics could also be given if the patient has infections or suppuration, usually followed by surgeries. However, drug-induced complications and toxicity should be kept in mind.

## Introduction and background

Crohn’s disease (CD) and ulcerative colitis (UC) are inflammatory bowel diseases (IBD) that cause chronic inflammation and damage in the gastrointestinal (GI) tract. Crohn’s disease can affect any part of the gastrointestinal tract, from the mouth to the anus. However, UC is limited to the large intestine (colon) and the rectum [1]. The prevalence is high in developed countries with 322 and 505/100,000 per year for CD and UC, respectively [2]. The key factors responsible for IBD include genetic components, environmental elements, microbial flora, and immune responses [3]. IBD is a systemic disease, and extraintestinal manifestations (EIM) are proof that IBD is not only limited to the gut. They most frequently affect joints, skin, or eyes, but can also less frequently involve other organs such as the liver, lungs, or pancreas [4].

EIMs of IBD have been implicated in all the anatomic sites of the pulmonary tree, and include airway inflammation of the bronchi and bronchioles (bronchiolitis, bronchiectasis, chronic bronchitis, and granulomatous bronchiolitis) and parenchymal inflammation (such as organizing pneumonia, interstitial pneumonia, lymphocytic interstitial pneumonia, eosinophilic pneumonitis, and granulomatous interstitial lung disease) [5]. Bronchial inflammation and suppuration are the most common manifestations of pulmonary involvement in IBD and include chronic bronchitis and bronchiectasis in which bronchial dilatation is visualized on chest X-ray or CT scan [6].

The involvement of the respiratory system in IBD, which can range from a simple defect of pulmonary function without symptoms, to fibrosing alveolitis with a greater risk of mortality, is relatively rare but sometimes potentially harmful [6]. Therefore, following the importance and need, the purpose of this study is to learn more about pulmonary manifestations of IBD.

Background

Beyond the bowel spread of IBD has always been a core discussion in literature over the decades. Studies predict around 21%-47% of the population with IBD develops extraintestinal manifestations [6]. Among these extraintestinal manifestations, pulmonary manifestations are variable, spanning from small to large airway diseases to parenchymal lung diseases, like interstitial pneumonitis, panbronchiolitis, bronchiolitis obliterans organizing pneumonia, inflammatory tracheal stenosis, serositis, apical fibrosis, Langerhans’s cell histiocytosis, sarcoidosis, and conditions resembling Wegener’s granulomatosis, and rarely thromboembolic complications, pulmonary vasculitis, pleural diseases, enteric-pulmonary fistulas or pulmonary function test abnormalities [6,7]. With a broad spectrum of presentation like low- or high-grade fever, chills, night sweats, mild to moderate cough, shortness of breath, pleuritic chest pain, headache, sinus drainage, and sometimes accidental abnormal radiographic finding on routine evaluation in an asymptomatic patient [8]. Imaging studies in patients with IBD have shown varying degrees of findings like an accidental finding of bilateral opacities and infiltrates in an asymptomatic patient, ill-defined, scattered nodules with cavitation on high-resolution computed tomography (HRCT). Radiologically, some patients showed nodular infiltrates bilaterally, some showed diffuse infiltrates, and some showed a mass. Bronchoscopy and thoracoscopies showed diffused lung appearance and a picture of interstitial pneumonitis with granulomatous changes with or without abscesses and necrotic debris. In another study on lung biopsies of patients having IBD, who were on mesalamine for years, showed chronic bronchiolitis with non-necrotizing granulomatous lesions, organizing pneumonia with focal granulomatous features, and interstitial pneumonitis with rare giant cells [9]. The most prevalent findings on pulmonary function tests (PFTs) were a decrease in forced expiratory volume in 1s, forced expiratory flow, FEV1/FVC, and transfer coefficient for carbon monoxide (DLCO), DLCO/ alveolar volume [10]. Patients with normal standard physiological pulmonary tests have been shown to have mosaic perfusion and air trapping on HRCT, suggestive of bronchiolitis obliterans and a pattern of branching linear opacities like a “tree in bud” appearance, suggestive of bronchiectasis with mucoid secretions [11]. However, gram staining and cultures were negative. These life-threatening symptoms and findings of pulmonary diseases are often overlooked by both pulmonologists and gastroenterologists. Thus, we should emphasize the importance of such investigations in patients with IBD, with or without any respiratory-related symptoms, to detect the earliest dysfunction and pulmonary abnormality [8,9]. So, physicians should take a broader approach in tackling patients with IBD, with or without clinical or physiological pulmonary manifestation.

Respiratory impairment can present decades after the presentation of bowel disease [11]. It is important to detect and treat IBD-related pulmonary manifestations early in the course of the disease before permanent destruction and irreversible impairment occurs [10]. This cross-talk between gut and lungs is still ambiguous. Where pulmonary impairment in patients with gut pathology is in the hype, it has also been found that patients with chronic obstructive pulmonary disease (COPD) or lung pathology are also at more risk to develop IBD [9, 12]. Finding a high prevalence of airway diseases in IBD patients and new-onset IBD in patients with respiratory diseases strongly support the hypothesis that the interplay between the two is mutual, and is two-way. Inappropriate immune response to microbiota may trigger the inflammation process in both systems. Dysregulation of the protease system is found in both IBD and COPD. Dysregulation in the immune cell homing system is a common mechanism found in all IBD, COPD, bronchiectasis, and asthma. All these common mechanisms can be a key feature for this association [13]. Several studies have shown a high prevalence of atopy in IBD patients. Overlapping allergies have been found in both gut and lung disorders [14]. A large study by Bernstein reported a high frequency of asthma in IBD patients [15].

Treatment strategies of these associated pulmonary symptoms depend upon the pattern or part of involvement. Steroids, in general, have shown to markedly decrease both, the extent and severity of symptoms [6,8]. A very recent study held in the year 2020 reported the first case of corona virus disease 2019 (COVID-19) in an elderly patient presenting with a history of Ulcerative colitis for three years. The patient was on mesalamine maintenance therapy when treated with corticosteroid for COVID symptoms, showed no mortality benefit. So, the International organization for the study of IBD (IOIBD) recommended the use of thiopurines and biologics whilst decreasing the use of corticosteroids [16].

The aim behind creating this review article was to highlight the cross talk between gut and lung, the possible mechanisms responsible for this association, and once again to emphasize the importance of a broader approach in IBD patients, early recognition, and treatment of bowel-related respiratory manifestations. 

This article also brings attention to the fact that the association between the two systems is two-way, so further researches should be done to declare the strong evidence that makes the foundation behind the correlation between these two major systems. Despite adequate literature on this association, there’s still room for further experimental studies on given pieces of evidence to bring current data into knowledge.

This review article will also serve as a ground for further studies on correlation with other systemic comorbidities associated with the involvement of these two systems. This might help the researchers to draw a new conclusion, or help them to identify potential areas to explore next. Also, talking about this gut-lung cross-talk and massive literature on their association generates a thought of correlating COVID-19 and IBD and their outcomes in further researches. 

## Review

Airway diseases

Strong correlations have been found between IBD and proximal or distal airway diseases like bronchiectasis, chronic bronchitis, bronchiolitis, subglottic stenosis, tracheobronchitis, and chronic bronchial suppuration. Airway and gut can be interlinked through their common embryological basis and same structural and physiological features, having the same columnar epithelium with mucous glands, goblet cells, and highly vascularized lumen [17]. However, the pathophysiology behind this disease correlation is still unknown [12]. Some researchers blame the same mucosal immunology of both the systems to be responsible for this correlation that makes bronchi hyper-reactive in response to active IBD [18]. The cross-link between the two systems is a multi-faceted process and involves multiple pathways [17]. Often airway involvement presents as the first symptom in an already existing chronic IBD for years and is the most common extraintestinal manifestation related to IBD [13]. These symptoms may exacerbate the mucosal pathology of the gut, and sometimes occur during the remission period, or even get worse after colectomy or ileostomy. So, no direct interlink was found between the severity and course of both the diseases [19]. Vice versa, increased prevalence of IBD has been found in patients with COPD than in the general population [12].

The majority of large and small airway pathologies showed a strong association with UC, whereas colo-pulmonary and tracheoesophageal fistulas are more commonly found with CD. Case reports and series have published a broad spectrum of pulmonary manifestations ranging from the larynx to pleura with bronchial inflammation being the most common manifestation in patients with UC with reported cases of bronchiectasis in 66% of the population with IBD. Bronchiectasis presents as an unexplained chronic cough with sputum that does not respond to antibiotics and shows bronchial dilatation on CT scan and chest X-ray. Bronchial biopsies show squamous cell metaplasia infiltrated by neutrophils, lymphocytes, and plasma cells. The second most common large airway pulmonary manifestation, after bronchiectasis, is chronic bronchitis that can be differentiated by the extent and degree of severity [20]. Patient with central airway involvement presents with symptoms like wheezing, difficult breathing, cough with or without sputum, pleuritic chest pain and limitation to work and exercise. Asthma is frequently reported [21]. Small airway diseases are less frequent, but present early in the span of disease and occur mostly in young patients. High-resolution CT has increased the diagnosis of small airway diseases by detecting early, minimal histopathological dysfunction. CT scan shows bronchial wall thickening, ground-glass nodules, mucoid impaction, and mosaic pattern due to air trapping. Bronchiolitis, mainly involving the intralobular and transition small airways, is fibrosing in nature and potentially life-threatening causing airway narrowing and distortion, thus completely obliterating the airways in the long run [22]. Steroids, both inhalational and systemic, have shown to bring marked declines in both severity and extent of IBD-associated pulmonary diseases and improve the symptoms to much extent.

Parenchymal diseases

The involvement of parenchyma in IBD is not very frequent and mostly happens late in the course of the disease. Female predominance has been seen, with the younger population at relative risk, and the older population at the absolute risk of developing pneumonia. Studies suggest a strong prediction of drugs being the main cause of causing parenchymal distortion, particularly immunosuppressants, and are mostly associated with UC than CD [6]. A study shows pulmonary pathology to be the most common cause of death in patients with IBD, infectious complications in particular. A study in the USA shows Community-acquired pneumonia to be the leading cause of death in IBD patients. Literature declared the patients with IBD to be the most vulnerable to develop pneumonia, particularly those on corticosteroid and anti-TNF agent [23]. Cryptogenic Organizing pneumonia (COP), also called bronchiolitis obliterans with organizing pneumonia (BOOP), was found to be the most common non-infectious IBD-related parenchymal disease. BOOP can be a result of inhalational injury or can be from the post-infection origin. Imaging studies show ground-glass opacities and multifocal patchy consolidation. Necrobiotic lung nodules have also been noticed [7,8]. BOOP shows a strong association with other autoimmune pathologies like Wegener granulomatosis, rheumatoid arthritis, and lupus. The infectious origin should always be excluded because presentation with necrotic nodule is unresponsive to antibiotics.

Interstitial diseases

IBD-related interstitial diseases are mainly a result of the drug-induced reaction of mesalamine and sulfasalazine causing eosinophilic pneumonia and interstitial pneumonitis. A rare case of fibrosing alveolitis has also been reported [6]. Pulmonary eosinophilia is a diagnosis of exclusion with a large percentage of eosinophils in BAL (bronchoalveolar lavage) fluid.

Five components should be seen before labeling any pulmonary manifestation as eosinophilic pneumonia.

(1) Rule out all other lung pathologies through history and imaging.

(2) There should be a drug history

(3) BAL fluid shows increased eosinophil count, and imaging findings should be consistent with eosinophilic pneumonitis.

(4) Withdrawal of culprit drugs causes improved symptoms and remission.

(5) Time course should be consistent with drug-induced reaction.

Patients fulfilling all the criteria are labeled as having the definitive disease. One with four criteria labeled as “possible” disease, and with three called to be “suspicious” [24]. A study by Fireman et al. reported a high number of eosinophils in the sputum of patients having ulcerative colitis [25]. This hypothesis also supports the idea that eosinophils derived-mediators can be a reason involved in the pathophysiology of IBD and super-imposed respiratory allergic reactions [26]. 

In contrast to this, another case study with a rare presentation of interstitial lung disease (ILD) mimicking pulmonary fibrosing alveolitis in a patient with CD on mesalamine for 3 months reported worsening of interstitial lung disease on mesalamine withdrawal. The histopathological features were the thickening of alveoli with collagen deposition and lymphoid infiltration without any granulomatous lesion. Interestingly, this case was exceptional from granulomatous pulmonary lesions seen with CD [27]. A study shows that most interstitial diseases are either pulmonary vasculitis or more commonly granulomatous diseases [28]. Combination therapy of steroid and cyclophosphamide showed favorable outcomes and cause marked remission in disease and symptom progression [27].

Drug-induced complication 

Pulmonary pathologies in patients with IBD are either due to drug toxicity or immune-related or due to an unknown inflammatory linkage with the gut disease, which is still controversial in the literature. However, some diseases are shown to be due to drug-induced reactions after excluding all other possible causes of lung pathology. So, a physician should always take a broader approach in IBD patients with pulmonary manifestation, and should always keep drug toxicity in mind while excluding all the causes. There are more than 200 drugs known to cause pulmonary distortion [29]. Higher numbers of eosinophilic pneumonia have been reported in patients on mesalamine and sulfasalazine, drugs used in IBD. A rare number of pleural effusions and pulmonary fibrosis has also been noticed [8]. When sulfasalazine was the only drug of choice, drug-induced toxicity was rare, but well recognized with histologic patterns correlating with imaging and clinical findings of bronchiolitis obliterans with interstitial pneumonia. Mesalamine-related pulmonary pathology was very low. Recognizing the cause, either mesalamine-related or disease-related is still difficult, several criteria should be met before diagnosing it as drug-related. When no other cause can be found, histopathological examination shows drug-related hypersensitivity reaction. Symptoms get better or subside on drug withdrawal [29].

A case report has given very strong evidence of drug toxicity in an 18-year-old patient with ulcerative colitis who was on combination therapy of mesalamine and azathioprine for a year, and presented with high-grade fever and patchy shadows with ill-defined margin on chest X-ray, and was diagnosed as drug-induced eosinophilic pneumonitis [15]. Another case report shows the development of dry cough and fever in a patient with ulcerative colitis for 6 months and was on oral mesalamine. Bilateral infiltrates were found on chest radiography. Both the clinical manifestation and radiographic signs started improving soon after drug withdrawal. Bronchial biopsy results were consistent with the organizing stage of eosinophilic pneumonia [30].

## Conclusions

Pulmonary extraintestinal manifestation in Inflammatory bowel disease is very frequent. They have a range from mild decline in pulmonary function to fibrosing alveolitis, a disease with very high mortality. It is important to be fully aware of all symptoms and pulmonary diseases associated with IBD. Early identification of latent pulmonary involvement is important to prevent any complications. Multidisciplinary team is needed to start treatment early and avoid further complications
